# Supplementary material for: Tidewater cycle drives alpine glacial sediment plume geochemistry
Source: Nat Commun. 2025 Oct 22;16:9211. doi: 10.1038/s41467-025-64731-1 (PMC12546839; doi:10.1038/s41467-025-64731-1)
Supplement: Supplementary file 1 — Supplementary Information [file 41467_2025_64731_MOESM1_ESM.pdf]

Supplemental Material: 'Tidewater cycle drives alpine glacial sediment plume geochemistry'

## On the bedrock source of glacial weathering

Unable to directly sample the bedrock underlying both Aialik and Northwestern Glaciers, we used cobbles collected from individual fjord iceberg samples as representative of the bedrock composition, as cobbles carried by icebergs are physical transported pieces of bedrock sourced from beneath the respective glaciers.

Literature indicates that the predominant bedrock beneath the Harding Icefield is Paleocene-Eocene granitic rocks, with the presence of metasedimentary rocks present on the periphery of the fjords. To investigate the variations in bedrock composition between the two fjords, we examine the rare earth element (REE) compositions of each cobble. The REE composition of sediment, dust, and bedrock are frequently used to investigate source provenance variability as the patterns of REEs fractionate as a result of differences in geochemical composition[1,2]. Shifts in REE patterns generally indicate differences in sediment or bedrock provenance, and the REE composition of cobbles from each of the fjords are generally similar (Fig. S8) with the exception of a positive Eu anomaly and a negative Er anomaly in cobbles from Aialik Glacier with respect to cobbles from Northwestern Glacier. Positive Eu anomalies in granitic rocks can be associated with earlier plagioclase crystallization during magmatic differentiation[3], perhaps signifying slight differences in the timing of plagioclase mineral crystallization in the granitic rocks beneath Aialik versus Northwestern Glacier. Elements such as iron and manganese are considered trace elements in the mineral plagioclase[4] and the weathering of rocks with slightly different plagioclase abundances should therefore not strongly influence the Fe and Mn flux transported to the marine environment.

We acknowledge that our cobbles do show some compositional variability, and we also acknowledge that cobbles may not be truly representative of the bulk bedrock composition underlying each of the glaciers. Importantly, higher concentrations of Fe and Mn in the cobble from AG (Table S2) is not matched by a similarly high flux of Fe and Mn in the dissolved phase (Table S1), nor in the total amount of particulate matter in meltwater. We see an opposite trend – greater concentrations of dFe and dMn as well as total pFe and pMn found in NWG despite lower concentrations of these metals in the cobble. Other studies focusing on subglacial meltwater geochemistry highlight that water-rock interaction time, rather than subglacial bedrock geochemistry are the primary drivers of observed nutrient and elemental concentrations[5]. The driver of the chemical differences in the cobbles from each respective fjord analyzed here may be attributed to the timing of crystallization of plagioclase, for which Fe and Mn are minor components. As biotite is the aluminosilicate favored to chemically weather first in these systems[6], the difference in the modal abundance of biotite in the bedrock is more important in terms of the resulting dFe and dMn concentrations in glacial meltwater.

This suggests that other factors such as water-rock interaction time (subglacial weathering regime) rather than the underlying bedrock geology is the driving influence behind the observed trends in dissolved metal concentrations. We therefore consider the subtle shifts in underlying bedrock compositions of each fjord as not a strong influencer of the resulting Fe and Mn fluxes in the marine particulates.

## On the diagnosis of subglacial weathering and transport processes

The use of elemental ratios provides diagnostic information on the behavior during subglacial weathering and transport processes, including deposition within the fjord. Given that elemental ratios have been applied to other polar, marine, and riverine systems[7–10], we apply them similarly. The following lines of evidence are presented in support of our interpretation for greater chemical weathering and sediment transport in NWG, which are distinct from indicators of mechanical weathering:

First, a comparison of the La-to-Sm ratios of the refractory components of sediments is useful for determining the extent of chemical weathering[11]. We find that sediments in NWG have on-average higher values of La-to-Sm (Fig. S9), indicating more extensive chemical weathering processes at play in this fjord system, as heavy REEs are depleted in the products after extreme weathering relative to light REEs[11,12]. It is important to note that the La-to-Sm ratio in cobbles are similar between the fjords (Fig. S8) indicating La-to-Sm ratios of sediments result from modification of a similar bedrock source.

Second, despite a difference in the composition of the cobbles from each fjord, we see on-average elevated Fe chemical lability (expressed as a fraction of the total particulate Fe), indicating that glacier weathering is the dominant control on Fe lability. This interpretation is supported by the findings of a previous study which examined the dFe concentration in meltwater streams draining the Greenland ice sheet, across a large range of lithologies[5]. The conclusion of this study demonstrated that water-rock interaction time (residence time of sediments) in the subglacial environment is the dominant control of the concentrations of dissolved metals, despite a range of source rock geochemistry.

However, we review the accessory minerals of granitic systems and suggest the preferential loss of these minerals during chemical weathering in glacial systems. The order of crystallization of granitic rocks follows: accessory minerals, ferromagnesian minerals, lime-alkali feldspar, alkali feldspar, and finally quartz[13]. The addition of Fe during magmatic differentiation is due to the rapid crystallization of phyllosilicates, which for the Harding Icefield includes the dominant mineral, biotite. Chemical weathering of biotite is the likely source for the high dissolved Fe and Si concentrations observed in other subglacial systems[14,15], which has been documented to chemically weather and be transported over periods of 145-275 years in proglacial environments and during early stages of glacial retreat[16]. Only in the oldest sediments does silicate weathering dominate (once carbonates have been removed and requiring prolonged periods of water-rock interaction), whereas biotite weathering occurs rapidly in the subglacial environment[6]. Aluminum oxides are considered the ultimate product of chemical weathering of these aluminosilicates, and we show that NWG has on-average greater Al chemical lability, compared to AG. This chemical lability is attributed to the presence of Al-oxides in addition to possible scavenging of dAl onto Fe- and Mn-oxides[17,18]. In both fjords, Al is enriched in the labile particulate phase, relative to Ti, indicating a greater adsorbed fraction of Al as Al-oxides relative to Ti[17]. The degree to which Al is enriched in particulate matter as Al-oxides is greatest in NWG, indicating intense chemical weathering has occurred or is occurring.

Hydrodynamic sorting may also contribute to the observed trends, with certain elements associated with the heavy/light and coarse/fine phases. In recent investigations, the Al-to-Ti ratio is applied to investigate the loss of heavy minerals associated with preferential gravitational settling of coarse-sized grains which contain Ti (e.g., rutile) due to hydrodynamic sorting (e.g., Aarons et al., 2023; Bouchez et al., 2011)[10,19]. The refractory (detrital) fraction of the sediments within AG has greater Al-to-Ti ratios compared to those in NWG. Combined with a greater contribution of the small particle size fraction to the overall concentrations of both Al and Ti, we

infer that a higher degree of hydrodynamic sorting has occurred on AG suspended sediments. This is unsurprising since injection of sediment-laden subglacial sediments occurs in the subsurface, escapes intense sedimentation processes at the grounding line, and must rise to the fjord surface since it is relatively fresh and less buoyant than the ambient seawater[20,21].

## Impacts of subglacial mechanical and chemical weathering on Al-to-Ti ratios

To address the question of the relative impacts of chemical weathering and transport on the geochemistry of suspended sediments, we applied the Al-to-Ti ratio (Figure 4a). The Al-to-Ti ratio of sediments is a useful indicator of particle sorting, where coarse particles enriched in primary minerals such as Fe-Ti oxides (and characterized by low Al-to-Ti ratio) are preferentially lost during transport as a result of density driven sorting (e.g., Aarons et al., 2023; Bouchez et al., 2011)[10,19]. Secondly, the labile particulate Al-to-Ti ratio is a useful indicator of the degree of chemical weathering, whereby Al-oxides are the ultimate product of intense chemical weathering of aluminosilicates (high labile particulate Al-to-Ti ratio)[17]. For context, we report the expected sediment sources supplied to each glacier sediment plume, detrital Al-to-Ti ratios in fjord surface sediments, the percent contribution of fine particles to the total suspended particulate concentration, and the labile particulate Al-to-Ti ratio as an indicator of Al-oxides content.

In AG, with the presence of a subsurface buoyant meltwater plume, we expect subglacial sediments to be highly sorted with a high contribution to the surface sediment plume. We also expect that through vigorous mixing with surrounding seawater, the rising buoyant plume would resuspend and entrain marine sediments. Finally, melting icebergs would contribute directly to the surface signature, although concentrations are too small within ice to explain the high concentrations at the fjord surface (Figure 4a). We find AG refractory/detrital (total minus labile) Al-to-Ti ratio of sediments to be on-average 28±8 and 34±8 mol:mol for small (<5 µm) and large (>5 µm) particle fractions, respectively. Small particles contribute on-average 50% and 53% of the total particulate concentrations of Al and Ti, respectively (Table S2). The labile particulate Al-to-Ti ratios are on-average 24±21 and 18±8 for the small and large particle fractions, respectively (Figure 4b).

In NWG, subglacial meltwaters discharge directly to the fjord surface, with no opportunity for fluvial sorting to occur. Icebergs would contribute to the fjord surface metal concentrations, but likely only a minor contribution as the sediment plume contains orders of magnitude greater sediment fluxes. We find NWG refractory/detrital (total minus labile) Al-to-Ti ratio of sediments to be on-average 21±0.6 and 24±1 mol:mol for small (<5 µm) and large (>5 µm) particle fractions, respectively. Small particles contribute on-average 44% and 47% of the total particulate concentrations of Al and Ti, respectively. The labile particulate Al-to-Ti ratios are on-average 38±21 and 28±11 for the small and large particle fractions, respectively (Fig. 4b).

The purpose of interrogating the Al-to-Ti ratios in the refractory and labile fractions is to understand the intensity of subglacial weathering and transport processes at different stages of the TGC. The higher Al-to-Ti ratios in detrital particles from AG indicates a higher degree of particle sorting, where coarse grains are lost from suspension (settled/sedimented) during transport from the subglacial environment to the fjord surface. The higher degree of particle sorting is also reflected in a larger contribution of small particles to the total suspended sediments in AG, compared to NWG. Finally, as Al-oxides are encompassed within the chemical extraction for labile particulate trace metals, we observed NWG particles to contain a greater

contribution of Al-oxides compared to AG sediments, indicating that both particle size fractions have been extensively chemically weathered, albeit large variability exists between stations.

## REFERENCES


1. Gabrielli, P. *et al.* Ultra-low rare earth element content in accreted ice from sub-glacial Lake Vostok, Antarctica. *Geochim. Cosmochim. Acta* **73**, 5959–5974 (2009).
2. Gabrielli, P. *et al.* A major glacial-interglacial change in aeolian dust composition inferred from Rare Earth Elements in Antarctic ice. *Quat. Sci. Rev.* **29**, 265–273 (2010).
3. Dygert, N., Ustunisik, G. K. & Nielsen, R. L. Europium in plagioclase-hosted melt inclusions reveals mantle melting modulates oxygen fugacity. *Nat. Commun.* **15**, 3033 (2024).
4. Nakada, R., Sato, M., Ushioda, M., Tamura, Y. & Yamamoto, S. Variation of Iron Species in Plagioclase Crystals by X-ray Absorption Fine Structure Analysis. *Geochemistry, Geophys. Geosystems* **20**, 5319–5333 (2019).
5. Aciego, S. M., Stevenson, E. I. & Arendt, C. A. Climate versus geological controls on glacial meltwater micronutrient production in southern Greenland. *Earth Planet. Sci. Lett.* **424**, 51–58 (2015).
6. Anderson, S. P., Drever, J. I., Frost, C. D. & Holden, P. Chemical weathering in the foreland of a retreating glacier. *Geochim. Cosmochim. Acta* **64**, 1173–1189 (2000).
7. Sherrell, R. M., Annett, A. L., Fitzsimmons, J. N., Roccanova, V. J. & Meredith, M. P. A 'shallow bathtub ring' of local sedimentary iron input maintains the Palmer Deep biological hotspot on the West Antarctic Peninsula shelf. *Philos. Trans. R. Soc. A Math. Phys. Eng. Sci.* (2018). doi:10.1098/rsta.2017.0171
8. Annett, A. L. *et al.* Controls on dissolved and particulate iron distributions in surface waters of the Western Antarctic Peninsula shelf. *Mar. Chem.* (2017). doi:10.1016/j.marchem.2017.06.004
9. Forsch, K. O. *et al.* Seasonal dispersal of fjord meltwaters as an important source of iron and manganese to coastal Antarctic phytoplankton. *Biogeosciences* **18**, 6349–6375 (2021).
10. Aarons, S. M. *et al.* Titanium transport and isotopic fractionation in the Critical Zone. *Geochim. Cosmochim. Acta* **352**, 175–193 (2023).
11. Wei, G., Li, X.-H., Liu, Y., Shao, L. & Liang, X. Geochemical record of chemical weathering and monsoon climate change since the early Miocene in the South China Sea. *Paleoceanography* **21**, (2006).
12. Nesbitt, H. W. Mobility and fractionation of rare earth elements during weathering of a granodiorite. *Nature* **279**, 206–210 (1979).
13. Naney, M. T. & Swanson, S. E. The effect of Fe and Mg on crystallization in granitic systems. *Am. Mineral.* **65**, 639–653 (1980).
14. Pryer, H. V *et al.* The Influence of Glacial Cover on Riverine Silicon and Iron Exports in Chilean Patagonia. *Global Biogeochem. Cycles* **34**, e2020GB006611 (2020).
15. Schroth, A. W., Crusius, J., Chever, F., Bostick, B. C. & Rouxel, O. J. Glacial influence on the geochemistry of riverine iron fluxes to the Gulf of Alaska and effects of deglaciation. *Geophys. Res. Lett.* **38**, (2011).
16. Föllmi, K. B., Arn, K., Hosein, R., Adatte, T. & Steinmann, P. Biogeochemical weathering in sedimentary chronosequences of the Rhône and Oberaar Glaciers (Swiss

Alps): Rates and mechanisms of biotite weathering. *Geoderma* **151**, 270–281 (2009).
17. Kryc, K. A., Murray, R. W. & Murray, D. W. Al-to-oxide and Ti-to-organic linkages in biogenic sediment: relationships to paleo-export production and bulk Al/Ti. *Earth Planet. Sci. Lett.* **211**, 125–141 (2003).
18. Murray, R. W. & Leinen, M. Scavenged excess aluminum and its relationship to bulk titanium in biogenic sediment from the central equatorial Pacific Ocean. *Geochim. Cosmochim. Acta* **60**, 3869–3878 (1996).
19. Bouchez, J., Lupker, M., Gaillardet, J., France-Lanord, C. & Maurice, L. How important is it to integrate riverine suspended sediment chemical composition with depth? Clues from Amazon River depth-profiles. *Geochim. Cosmochim. Acta* **75**, 6955–6970 (2011).
20. Eidam, E. F., Nittrouer, C. A., Lundesgaard, Homolka, K. K. & Smith, C. R. Variability of Sediment Accumulation Rates in an Antarctic Fjord. *Geophys. Res. Lett.* (2019). doi:10.1029/2019GL084499
21. Syvitski, J. P. M. On the deposition of sediment within glacier-influenced fjords: Oceanographic controls. *Mar. Geol.* **85**, 301–329 (1989).

## FIGURES

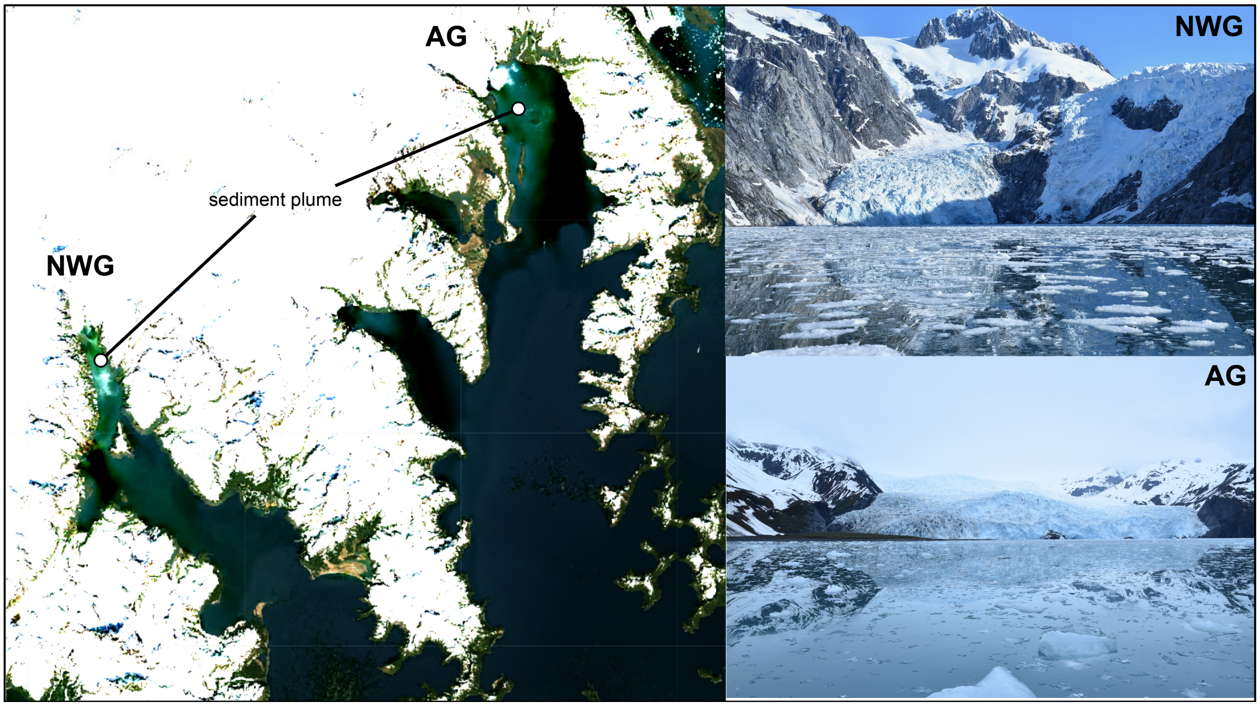


**Figure S1**. Landsat truecolor image of NWG (left) and AG (right) showing glacial ice in the inner fjords as well as glacial sediment plumes along the western flanks of both fjords. The satellite image was taken on May 9th, 2022.

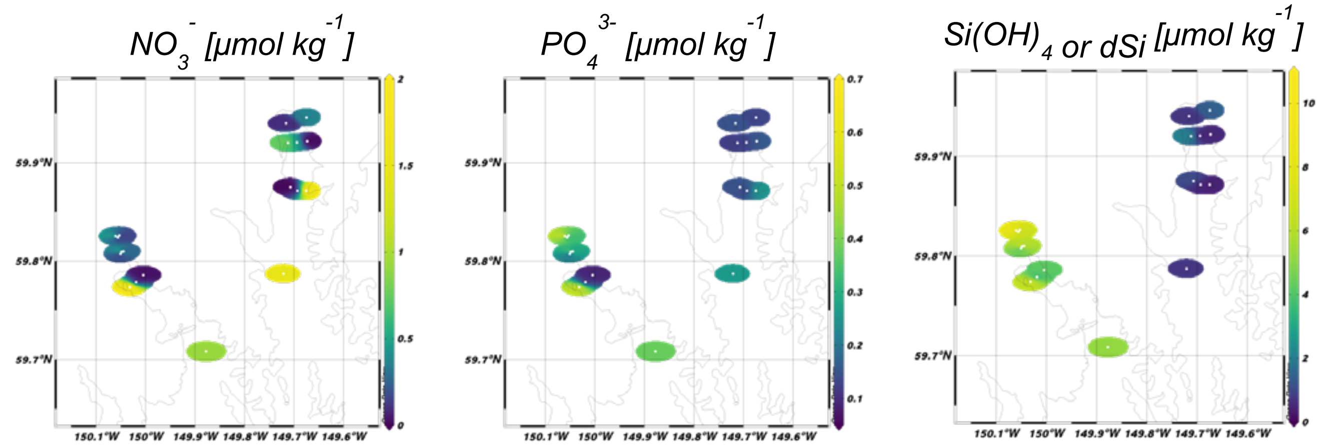


**Figure S2**. Surface concentrations of the macronutrients nitrate ($NO_3^-$), phosphate ($PO_4^{3-}$), and silicic acid (dSi). The grey outline is the coastline.

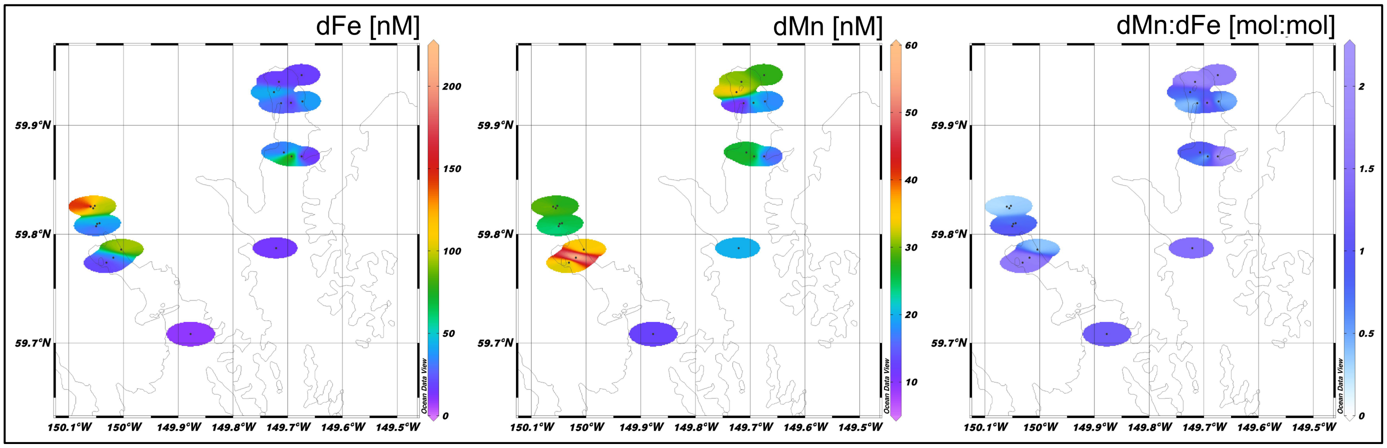


**Figure S3**. Surface concentrations of dissolved Fe and Mn. The ratio of surface dissolved Mn-to-Fe is also plotted. The grey outline is the coastline.

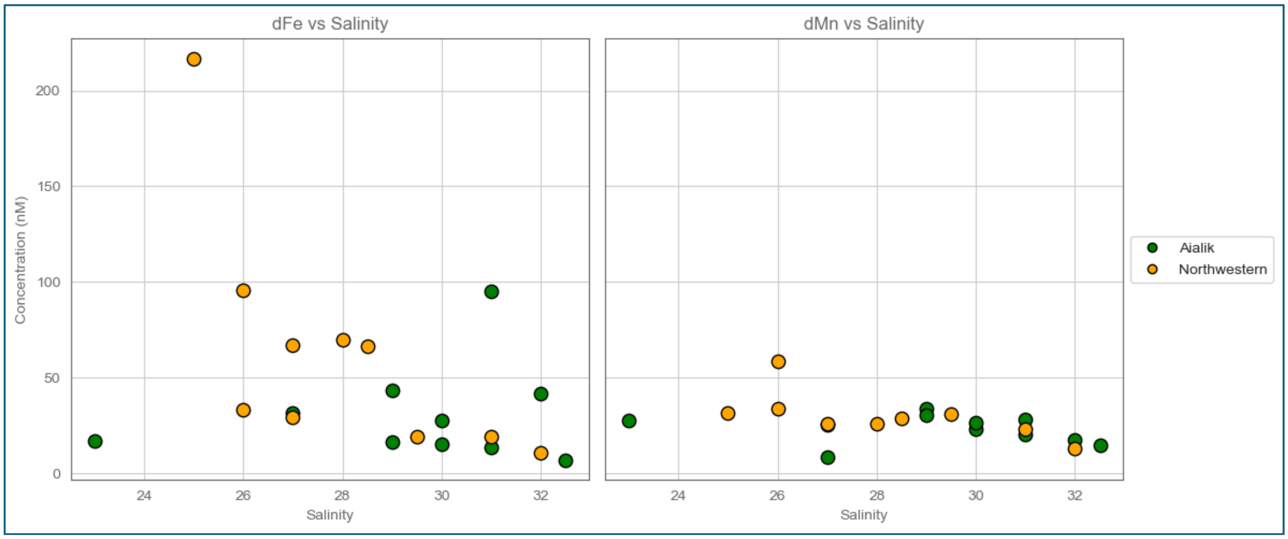

**Figure S4**. Dissolved Fe (left) and Mn (right) versus sea surface salinity.

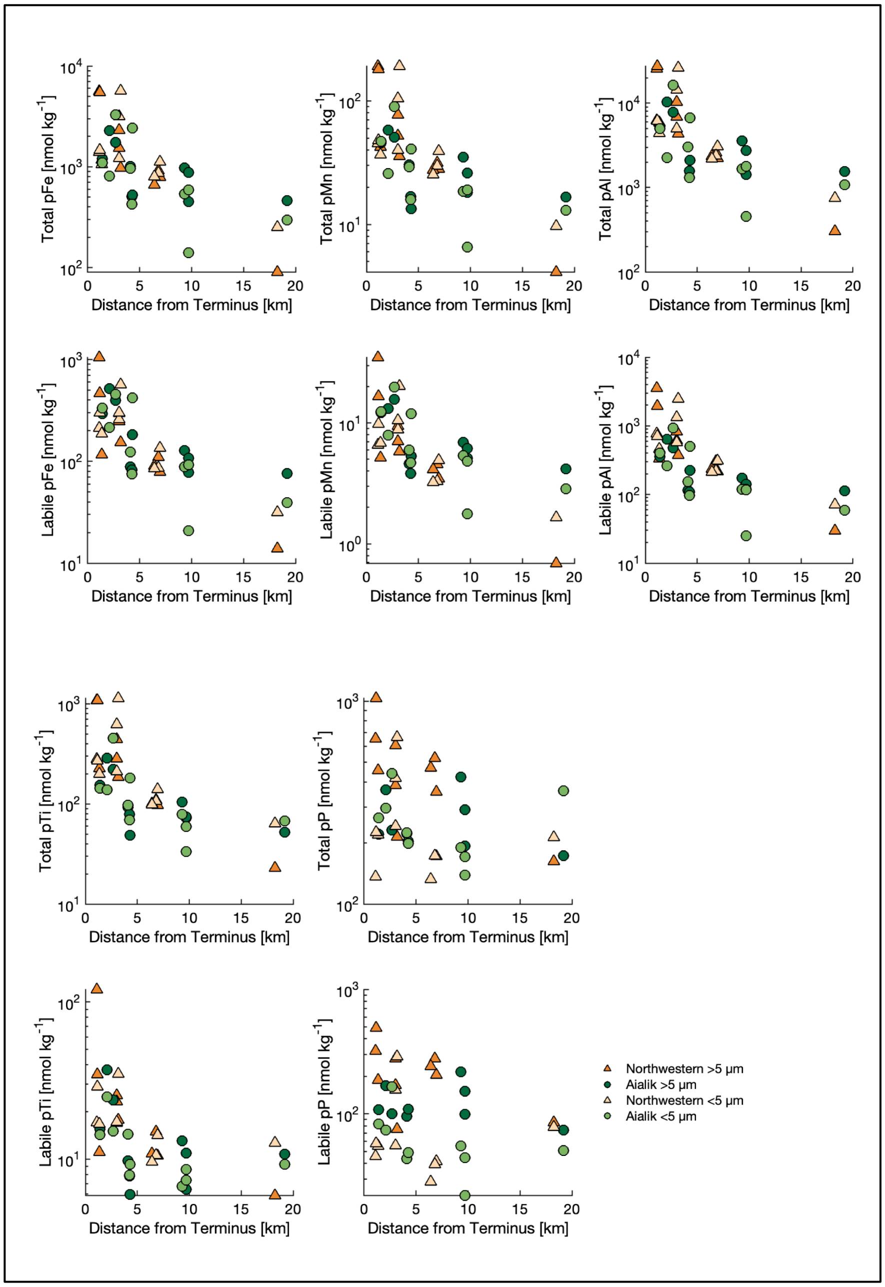


**Figure S5**. Concentrations of total and labile particulate trace metals (Fe, Mn, Al, Ti, and P) in suspended marine particles for NW (triangles) and Aialik (circles) fjords are plotted as the distance each station is from the glacier terminus.

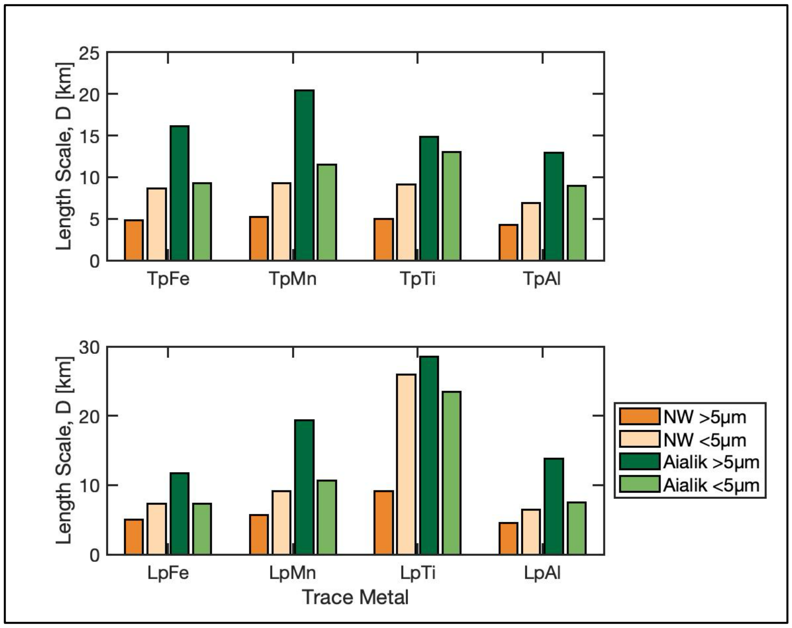


**Figure S6.** Length-scales ($D$) of total (top) and labile (bottom) particulate trace metal concentrations. The parameter $D$ comes from the exponential decay equation ($C_x = C_0 * e^{-x/D}$) fit to the measured concentrations ($C_x$) as a function of distance ($x$) from the glacier terminus.

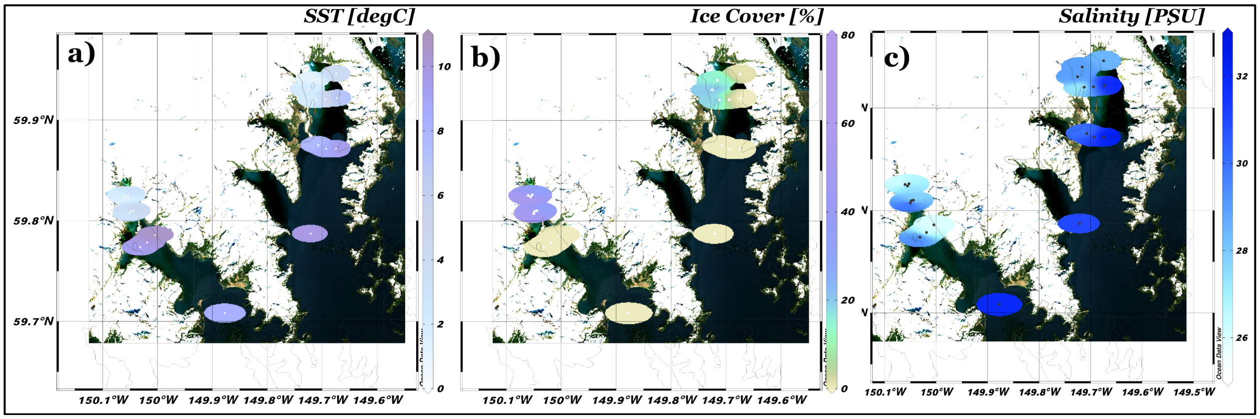


**Figure S7**. Surface plots showing the sea surface temperature (a), percentage of sea surface covered by icebergs (b), and surface salinity measured by a refractometer (c).

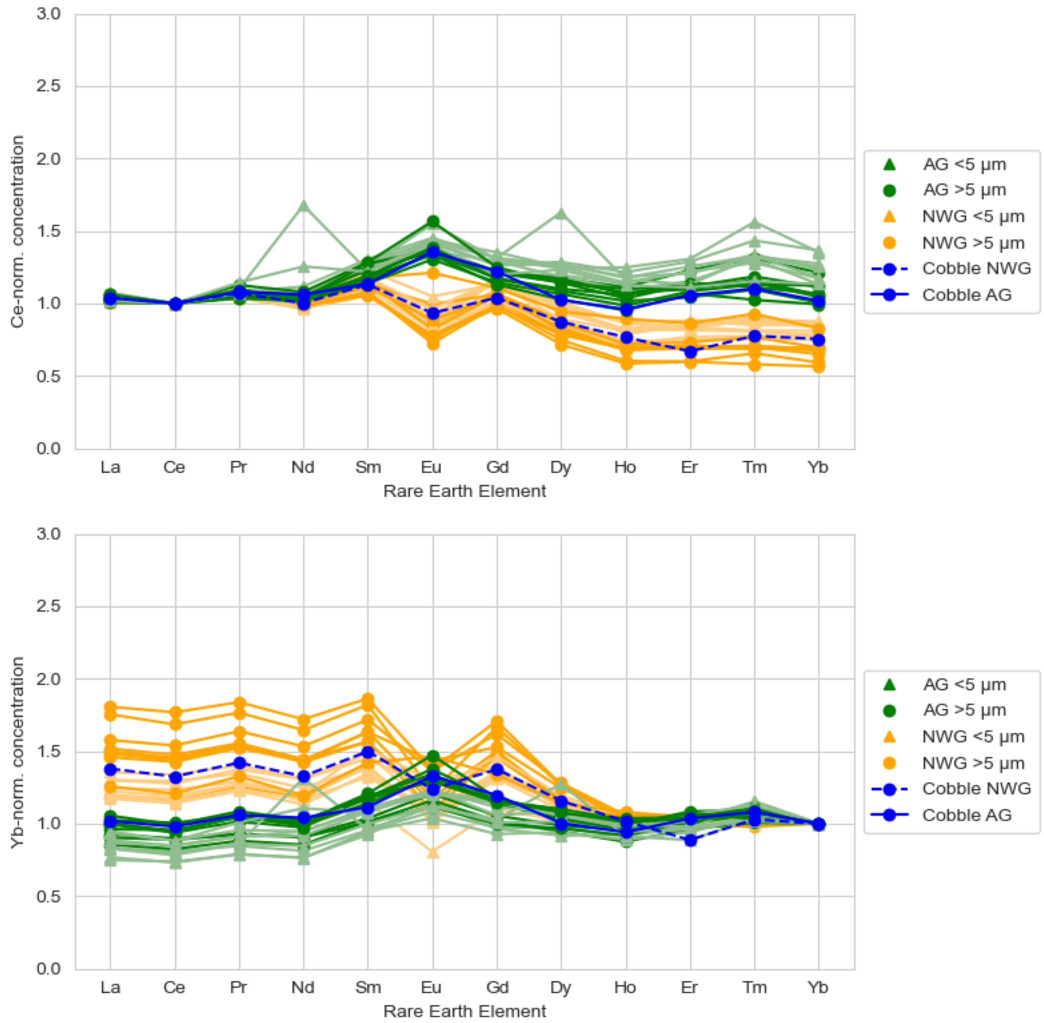


**Figure S8.** Rare Earth Element (REE) spider diagrams for detrital fjord sediments and cobbles from both fjords with the color corresponding to the fjord (green = AG; orange = NWG) or cobbles (blue). Triangles represent the fine particle (<5 µm) fraction, while circles are the large particle (>5 µm) fraction. Cerium-normalized concentrations (top figure) are often used to demonstrate variability in source provenance, while Yb-normalized concentrations (bottom figure) account for differences sediment concentration in driving the observed trends.

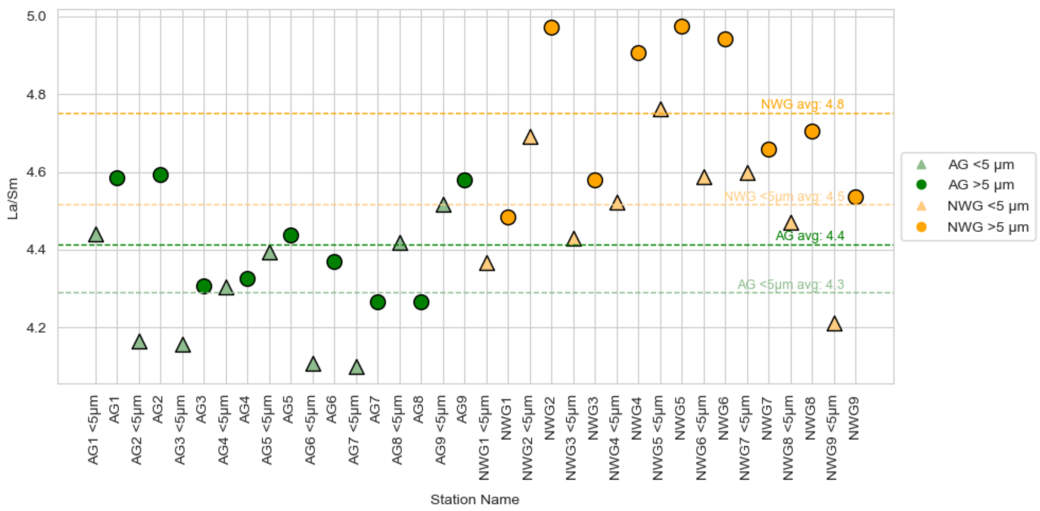


**Figure S9.** The La-to-Sm ratios (mol:mol), an indicator of the intensity of chemical weathering [11] is plotted for all stations. Horizontal lines represent the average La-to-Sm ratio (mol:mol) for each particle size class.

**Table S1**. Table of water column variables and soluble elemental data. Macronutrients at station 1 in Aialik Fjord were not measured.

| | Station | Latitude | Longitude | Ice Cover | Sea Surface Temperature | Salinity | Bottom Depth | Nitrate | Phosphate | dSi | dFe | dMn | dMn:dFe |
|---|---|---|---|---|---|---|---|---|---|---|---|---|---|
| | no. | [degrees N] | [degrees W] | [%] | [degC] | [PSU] | [m] | [μM] | [μM] | [μM] | [nM] | [nM] | [mol:mol] |
| Aialik Fjord | 1 | 59.93 | -149.73 | 20 | 2.1 | 29 | 112 | *na* | *na* | *na* | 43.66 | 34.11 | 0.78 |
| | 2 | 59.94 | -149.72 | 15 | 1.2 | 29 | 60 | 0.09 | 0.15 | 0.7 | 16.55 | 30.70 | 1.86 |
| | 3 | 59.95 | -149.67 | 1 | 4.3 | 23 | 59 | 0.36 | 0.13 | 1.4 | 16.71 | 27.91 | 1.67 |
| | 4 | 59.92 | -149.67 | 0 | 7.1 | 32 | 14 | 0 | 0.16 | 0.5 | 41.61 | 17.53 | 0.42 |
| | 5 | 59.92 | -149.69 | 1 | 5.2 | 30 | 145 | 0.45 | 0.13 | 0.3 | 15.24 | 23.34 | 1.53 |
| | 6 | 59.92 | -149.71 | 20 | 1.7 | 27 | 82 | 0.77 | 0.12 | 2.2 | 31.39 | 8.81 | 0.28 |
| | 7 | 59.88 | -149.71 | 0 | 7.7 | 30 | 10 | 0 | 0.15 | 1.1 | 27.55 | 26.56 | 0.96 |
| | 8 | 59.87 | -149.69 | 0 | 8.6 | 31 | 68 | 0 | 0.13 | 0.4 | 95.15 | 28.40 | 0.30 |
| | 9 | 59.87 | -149.67 | 0 | 9.5 | 32.5 | 11 | 1.87 | 0.26 | 0.4 | 7.03 | 14.69 | 2.09 |
| | 10 | 59.79 | -149.72 | 0 | 9.8 | 31 | 292 | 1.53 | 0.26 | 0.7 | 13.85 | 20.10 | 1.45 |
| Northwestern Fjord | 1 | 59.83 | -150.06 | 50 | 1.9 | 25 | 165 | 0.77 | 0.67 | 10.9 | 216.61 | 31.50 | 0.15 |
| | 2 | 59.82 | -150.06 | 75 | 0.2 | 28.5 | 182 | 0.01 | 0.23 | 5.4 | 66.67 | 28.75 | 0.43 |
| | 3 | 59.83 | -150.05 | 10 | 4.3 | 27 | 94 | 0.02 | 0.28 | 6.4 | 67.16 | 25.34 | 0.38 |
| | 4 | 59.81 | -150.04 | 5 | 5.8 | 27 | 117 | 0.01 | 0.22 | 5.7 | 29.61 | 26.01 | 0.88 |
| | 5 | 59.81 | -150.05 | 75 | 3.2 | 28 | 213 | 0.87 | 0.47 | 7.6 | 69.63 | 26.17 | 0.38 |
| | 6 | 59.81 | -150.05 | 50 | 2.5 | 31 | 212 | 0 | 0.14 | 3.3 | 19.14 | 22.91 | 1.20 |
| | 7 | 59.77 | -150.03 | 0 | 9.4 | 29.5 | 131 | 1.88 | 0.52 | 6.7 | 19.05 | 31.24 | 1.64 |
| | 8 | 59.78 | -150.02 | 0 | 10.1 | 26 | 130 | 0 | 0.09 | 3.7 | 33.46 | 58.44 | 1.75 |
| | 9 | 59.79 | -150.00 | 0 | 10.7 | 26 | 146 | 0.03 | 0.09 | 4.2 | 95.70 | 33.57 | 0.35 |
| | 10 | 59.71 | -149.88 | 0 | 8.3 | 32 | 161 | 0.93 | 0.38 | 5.2 | 10.55 | 12.94 | 1.23 |

**Table S2**. Table of particulate matter leach and digest data for seawater, icebergs, and cobbles. Column labels define the metal (Me) and components where ‘LpMe_0.4’ refers to the ‘labile particulate’ fraction less than 5 µm, but greater than 0.4 µm (small particles), while ‘TpMe’ refers to the ‘total particulate’ fraction greater than 5 µm (large particles). Cobbles were processed as powders and were not distinguished based on particle size. Iceberg concentration units are in nmol or µmol per kilogram of meltwater. Concentration units for cobbles are µmol per gram dry weight sediment (d.w.s.).

| | Station no. | Latitude [degrees N] | Longitude [degrees W] | LpAl_0.4 [nmol kg-1] | LpTi_0.4 [nmol kg-1] | LpFe_0.4 [nmol kg-1] | LpMn_0.4 [nmol kg-1] | LpP_0.4 [nmol kg-1] | TpAl_0.4 [µmol kg-1] | TpTi_0.4 [nmol kg-1] | TpFe_0.4 [nmol kg-1] | TpMn_0.4 [nmol kg-1] | TpP_0.4 [nmol kg-1] | TpAl:TpTi_0.4 [mol:mol] | LpAl:LpTi_0.4 [mol:mol] | TpFe:TpAl_0.4 [mol:mol] | LpFe:LpAl_0.4 [mol:mol] | TpMn:TpFe_0.4 [mol:mol] | LpMn:LpFe_0.4 [mol:mol] |
|---|---|---|---|---|---|---|---|---|---|---|---|---|---|---|---|---|---|---|---|
| Aialik Fjord | 1 | 59.930 | -149.725 | 933.20 | 15.09 | 457.00 | 19.91 | 164.58 | 16.40 | 454.42 | 3271.89 | 90.37 | 439.49 | 36.09 | 61.85 | 0.20 | 0.49 | 0.028 | 0.044 |
| | 2 | 59.940 | -149.716 | 402.24 | 14.34 | 332.93 | 12.49 | 83.18 | 4.97 | 142.91 | 1101.19 | 47.02 | 265.87 | 34.81 | 28.05 | 0.22 | 0.83 | 0.043 | 0.038 |
| | 3 | 59.946 | -149.675 | 263.43 | 24.97 | 214.78 | 7.93 | 73.82 | 2.26 | 138.26 | 813.52 | 25.91 | 297.56 | 16.35 | 10.55 | 0.36 | 0.82 | 0.032 | 0.037 |
| | 4 | 59.922 | -149.673 | 154.58 | 14.44 | 124.32 | 5.97 | 43.65 | 3.02 | 97.33 | 962.05 | 29.37 | 224.61 | 31.07 | 10.70 | 0.32 | 0.80 | 0.031 | 0.048 |
| | 5 | 59.921 | -149.694 | 96.60 | 7.92 | 75.63 | 4.70 | 48.84 | 1.30 | 68.96 | 426.15 | 15.94 | 198.95 | 18.90 | 12.19 | 0.33 | 0.78 | 0.037 | 0.062 |
| | 6 | 59.920 | -149.712 | 505.67 | 9.30 | 419.61 | 11.99 | *na* | 6.69 | 182.22 | 2427.30 | 40.95 | *na* | 36.72 | 54.36 | 0.36 | 0.83 | 0.017 | 0.029 |
| | 7 | 59.875 | -149.707 | 119.82 | 6.72 | 88.17 | 5.38 | 55.25 | 1.66 | 78.66 | 538.20 | 18.44 | 190.25 | 21.08 | 17.83 | 0.32 | 0.74 | 0.034 | 0.061 |
| | 8 | 59.871 | -149.693 | 117.20 | 7.37 | 92.60 | 4.84 | 44.49 | 1.79 | 59.24 | 591.98 | 19.01 | 171.10 | 30.29 | 15.91 | 0.33 | 0.79 | 0.032 | 0.052 |
| | 9 | 59.872 | -149.675 | 24.90 | 8.57 | 20.75 | 1.77 | 22.21 | 0.45 | 33.24 | 141.46 | 6.56 | 139.11 | 13.68 | 2.91 | 0.31 | 0.83 | 0.046 | 0.085 |
| | 10 | 59.787 | -149.721 | 58.48 | 9.28 | 39.22 | 2.86 | 50.86 | 1.08 | 67.63 | 298.20 | 13.01 | 361.97 | 16.01 | 6.30 | 0.28 | 0.67 | 0.044 | 0.073 |
| Northwestern Fjord | 1 | 59.825 | -150.060 | 770.31 | 17.07 | 211.92 | 6.61 | 45.77 | 6.27 | 281.04 | 1404.50 | 45.53 | 136.72 | 22.31 | 45.12 | 0.22 | 0.28 | 0.032 | 0.031 |
| | 2 | 59.824 | -150.056 | 460.59 | 16.81 | 187.99 | 6.84 | 55.19 | 4.39 | 198.72 | 1057.68 | 36.89 | 219.46 | 22.09 | 27.40 | 0.24 | 0.41 | 0.035 | 0.036 |
| | 3 | 59.826 | -150.053 | 702.83 | 28.87 | 301.59 | 9.85 | 57.83 | 6.20 | 271.60 | 1465.64 | 47.83 | 226.38 | 22.84 | 24.34 | 0.24 | 0.43 | 0.033 | 0.033 |
| | 4 | 59.810 | -150.044 | 578.47 | 16.97 | 259.48 | 8.81 | 55.73 | 4.98 | 212.45 | 1217.89 | 39.87 | 241.58 | 23.45 | 34.09 | 0.24 | 0.45 | 0.033 | 0.034 |
| | 5 | 59.810 | -150.048 | 1333.14 | 17.27 | 299.86 | 10.55 | 155.52 | 14.36 | 623.59 | 3151.11 | 105.07 | 417.93 | 23.02 | 77.20 | 0.22 | 0.22 | 0.033 | 0.035 |
| | 6 | 59.807 | -150.050 | 2493.78 | 34.82 | 568.08 | 20.14 | 287.82 | 26.39 | 1143.38 | 5687.91 | 191.97 | 664.96 | 23.08 | 71.62 | 0.22 | 0.23 | 0.034 | 0.035 |
| | 7 | 59.774 | -150.032 | 310.84 | 14.22 | 135.01 | 4.92 | 41.61 | 3.05 | 140.45 | 1126.32 | 39.42 | 172.01 | 21.74 | 21.86 | 0.37 | 0.43 | 0.035 | 0.036 |
| | 8 | 59.779 | -150.019 | 221.05 | 10.59 | 86.22 | 3.30 | 39.42 | 2.39 | 107.46 | 869.78 | 29.81 | 173.47 | 22.29 | 20.88 | 0.36 | 0.39 | 0.034 | 0.038 |
| | 9 | 59.786 | -150.005 | 212.96 | 9.66 | 85.75 | 3.23 | 28.46 | 2.18 | 98.68 | 796.80 | 25.31 | 132.66 | 22.14 | 22.05 | 0.36 | 0.40 | 0.032 | 0.038 |
| | 10 | 59.708 | -149.878 | 71.70 | 12.67 | 31.56 | 1.66 | 78.05 | 0.75 | 63.40 | 251.26 | 9.70 | 213.36 | 11.84 | 5.66 | 0.33 | 0.44 | 0.039 | 0.053 |
| Icebergs | 1 | | | 0.64 | 0.015 | 0.24 | 0.004 | 0.39 | 36.41 | 1.57 | 7.13 | 0.14 | 1.76 | 23.12 | 41.61 | 0.20 | 0.38 | 0.019 | 0.017 |
| | 2 | | | 1.07 | 0.034 | 0.58 | 0.012 | 0.30 | 29.87 | 1.24 | 7.46 | 0.19 | 6.11 | 24.04 | 31.60 | 0.25 | 0.55 | 0.026 | 0.021 |
| | 3 | | | 0.15 | 0.009 | 0.12 | 0.001 | 0.44 | 21.31 | 0.52 | 5.79 | 0.10 | 2.63 | 40.76 | 16.86 | 0.27 | 0.82 | 0.018 | 0.007 |
| | 4 | | | 1.15 | 0.091 | 1.81 | 0.047 | 4.61 | 19.60 | 0.83 | 30.69 | 0.46 | 93.01 | 23.56 | 12.73 | 1.57 | 1.57 | 0.015 | 0.026 |
| | 5 | | | 8.15 | 0.113 | 2.99 | 0.086 | 5.67 | 237.10 | 5.33 | 47.41 | 1.07 | 21.89 | 44.51 | 72.17 | 0.20 | 0.37 | 0.023 | 0.029 |
| | | | | LpAl [µmol g-1 d.w.s.] | LpTi [µmol g-1 d.w.s.] | LpFe [µmol g-1 d.w.s.] | LpMn [µmol g-1 d.w.s.] | LpP [µmol g-1 d.w.s.] | TpAl [µmol g-1 d.w.s.] | TpTi [µmol g-1 d.w.s.] | TpFe [µmol g-1 d.w.s.] | TpMn [µmol g-1 d.w.s.] | TpP [µmol g-1 d.w.s.] | TpAl:TpTi [mol:mol] | LpAl:LpTi [mol:mol] | TpFe:TpAl [mol:mol] | LpFe:LpAl [mol:mol] | TpMn:TpFe [mol:mol] | LpMn:LpFe [mol:mol] |
| Cobbles | 1 | | | 51.20 | 1.55 | 26.36 | 4.14 | 15.52 | 3366.69 | 199.80 | 1019.07 | 33.91 | 57.09 | 16.85 | 33.07 | 0.30 | 0.51 | 0.033 | 0.16 |
| | 2 | | | 49.15 | 0.37 | 13.90 | 1.85 | 10.06 | 3180.19 | 144.43 | 424.88 | 18.16 | 35.55 | 22.02 | 131.66 | 0.13 | 0.28 | 0.043 | 0.13 |

**Table S2 (continued).** Table of particulate matter leach and digest data for seawater, icebergs, and cobbles. Column labels define the metal (Me) and components where ‘LpMe_0.4’ refers to the ‘labile particulate’ fraction less than 5 µm, but greater than 0.4 µm (small particles), while ‘TpMe’ refers to the ‘total particulate’ fraction greater than 5 µm (large particles). Cobbles were processed as powders and were not distinguished based on particle size. Iceberg concentration units are in nmol or µmol per kilogram of meltwater. Concentration units for cobbles are µmol per gram dry weight sediment (d.w.s.).

| | Station no. | LpAl [nmol kg-1] | LpTi [nmol kg-1] | LpFe [nmol kg-1] | LpMn [nmol kg-1] | LpP [nmol kg-1] | TpAl [µmol kg-1] | TpTi [nmol kg-1] | TpFe [nmol kg-1] | TpMn [nmol kg-1] | TpP [nmol kg-1] | TpAl:TpTi [mol:mol] | LpAl:LpTi [mol:mol] | TpFe:TpAl [mol:mol] | LpFe:LpAl [mol:mol] | TpMn:TpFe [mol:mol] | LpMn:LpFe [mol:mol] |
|---|---|---|---|---|---|---|---|---|---|---|---|---|---|---|---|---|---|
| Aialik Fjord | 1 | 481.19 | 23.76 | 398.75 | 15.65 | 99.85 | 7.78 | 221.64 | 1741.59 | 50.75 | 231.25 | 35.08 | 20.26 | 0.22 | 0.83 | 0.029 | 0.039 |
| | 2 | 358.49 | 15.60 | 294.08 | 12.13 | 107.93 | 5.31 | 154.66 | 1201.46 | 45.70 | 221.27 | 34.31 | 22.98 | 0.23 | 0.82 | 0.038 | 0.041 |
| | 3 | 634.18 | 37.01 | 521.18 | 13.12 | 169.26 | 10.41 | 288.77 | 2294.57 | 58.23 | 365.12 | 36.04 | 17.14 | 0.22 | 0.82 | 0.025 | 0.025 |
| | 4 | 114.37 | 9.79 | 88.29 | 4.59 | 95.36 | 3.04 | 93.34 | 1011.50 | 30.48 | 221.98 | 32.59 | 11.69 | 0.33 | 0.77 | 0.030 | 0.052 |
| | 5 | 109.55 | 7.79 | 82.82 | 3.82 | 108.77 | 1.56 | 79.09 | 508.78 | 16.79 | 203.74 | 19.78 | 14.07 | 0.33 | 0.76 | 0.033 | 0.046 |
| | 6 | 223.47 | 5.96 | 182.26 | 5.35 | *na* | 2.12 | 48.52 | 523.70 | 13.40 | *na* | 43.63 | 37.51 | 0.25 | 0.82 | 0.026 | 0.029 |
| | 7 | 174.95 | 13.09 | 127.78 | 6.91 | 218.21 | 3.57 | 104.06 | 977.31 | 34.98 | 423.46 | 34.35 | 13.37 | 0.27 | 0.73 | 0.036 | 0.054 |
| | 8 | 141.56 | 6.42 | 108.16 | 5.10 | 99.46 | 2.74 | 72.78 | 874.90 | 26.20 | 193.65 | 37.70 | 22.04 | 0.32 | 0.76 | 0.030 | 0.047 |
| | 9 | 118.92 | 10.93 | 78.03 | 6.17 | 152.46 | 1.42 | 74.17 | 451.66 | 18.17 | 291.65 | 19.20 | 10.88 | 0.32 | 0.66 | 0.040 | 0.079 |
| | 10 | 114.03 | 10.81 | 76.46 | 4.15 | 74.42 | 1.55 | 52.00 | 460.02 | 16.61 | 172.55 | 29.86 | 10.55 | 0.30 | 0.67 | 0.036 | 0.054 |
| Northwestern Fjord | 1 | 3545.86 | 120.55 | 1055.93 | 35.08 | 320.27 | 25.70 | 1085.80 | 5588.58 | 191.93 | 653.89 | 23.67 | 29.41 | 0.22 | 0.30 | 0.034 | 0.033 |
| | 2 | 333.06 | 11.13 | 116.79 | 5.15 | 187.39 | 5.89 | 225.66 | 1171.01 | 42.20 | 456.91 | 26.08 | 29.94 | 0.20 | 0.35 | 0.036 | 0.044 |
| | 3 | 1958.87 | 34.53 | 467.60 | 16.70 | 491.39 | 27.64 | 1079.53 | 5500.66 | 181.19 | 1035.95 | 25.60 | 56.74 | 0.20 | 0.24 | 0.033 | 0.036 |
| | 4 | 618.93 | 25.43 | 248.80 | 9.47 | 277.05 | 6.86 | 282.55 | 1535.74 | 52.49 | 606.46 | 24.28 | 24.34 | 0.22 | 0.40 | 0.034 | 0.038 |
| | 5 | 826.54 | 23.38 | 298.43 | 7.05 | 169.80 | 10.34 | 444.22 | 2323.16 | 77.12 | 384.74 | 23.28 | 35.36 | 0.22 | 0.36 | 0.033 | 0.024 |
| | 6 | 374.78 | 17.73 | 153.84 | 5.80 | 75.82 | 4.38 | 185.00 | 976.95 | 35.62 | 214.74 | 23.69 | 21.14 | 0.22 | 0.41 | 0.036 | 0.038 |
| | 7 | 217.95 | 10.50 | 78.75 | 3.48 | 205.00 | 2.22 | 96.89 | 786.50 | 28.13 | 358.07 | 22.87 | 20.76 | 0.35 | 0.36 | 0.036 | 0.044 |
| | 8 | 305.09 | 14.92 | 109.63 | 4.57 | 277.93 | 2.60 | 109.18 | 925.55 | 31.66 | 523.41 | 23.78 | 20.45 | 0.36 | 0.36 | 0.034 | 0.042 |
| | 9 | 235.40 | 10.83 | 90.53 | 4.12 | 240.45 | 2.35 | 100.98 | 664.37 | 27.60 | 469.13 | 23.31 | 21.74 | 0.28 | 0.38 | 0.042 | 0.045 |
| | 10 | 29.87 | 5.89 | 13.88 | 0.69 | 85.35 | 0.30 | 22.78 | 90.86 | 4.13 | 162.00 | 13.36 | 5.07 | 0.30 | 0.46 | 0.045 | 0.050 |
| Icebergs | 1 | 6.83 | 0.09 | 2.59 | 0.049 | 1.13 | 349.71 | 18.68 | 95.84 | 2.24 | 7.03 | 18.72 | 77.95 | 0.27 | 0.38 | 0.023 | 0.019 |
| | 2 | 3.99 | 0.11 | 3.21 | 0.071 | 0.51 | 232.77 | 6.47 | 64.30 | 1.69 | 3.40 | 36.00 | 37.94 | 0.28 | 0.81 | 0.026 | 0.022 |
| | 3 | 4.54 | 0.05 | 0.96 | 0.014 | 0.63 | 655.80 | 20.81 | 106.74 | 3.42 | 8.45 | 31.51 | 84.07 | 0.16 | 0.21 | 0.032 | 0.015 |
| | 4 | 124.43 | 2.85 | 62.98 | 5.135 | 67.54 | 7078.78 | 237.26 | 1700.46 | 148.01 | 644.83 | 29.84 | 43.62 | 0.24 | 0.51 | 0.087 | 0.082 |
| | 5 | 1695.47 | 36.60 | 583.81 | 35.150 | 328.97 | 93647.92 | 3818.60 | 17428.46 | 1159.38 | 2834.00 | 24.52 | 46.33 | 0.19 | 0.34 | 0.067 | 0.060 |
