# Supplementary material for: Tidewater cycle drives alpine glacial sediment plume geochemistry
Source: Nat Commun. 2025 Oct 22;16:9211. doi: 10.1038/s41467-025-64731-1 (PMC12546839; doi:10.1038/s41467-025-64731-1)
Supplement: Supplementary file 2 — Description of Additional Supplementary Information [file 41467_2025_64731_MOESM2_ESM.pdf]

Supplementary Dataset 1

The Excel spreadsheet contains rare earth element concentrations measured in individual suspended marine particle samples and cobbles and referred to in the Supplementary Material.
